# A Holistic Approach to Managing Secondary Dysphagia Following Prolonged Intubation and Tracheostomy: A Case Report

**DOI:** 10.7759/cureus.34620

**Published:** 2023-02-04

**Authors:** Abhishek Bharadwaj, Praveen K Neema, Habib Md R Karim, Manas P Borthakur, Monica Khetarpal

**Affiliations:** 1 Anesthesiology, Critical Care, and Pain Medicine, All India Institute of Medical Sciences, Raipur, IND; 2 Cardiac Anesthesiology, Amrita Institute of Medical Sciences and Research Centre, Kochi, IND; 3 Anesthesiology and Critical Care, Sarathi Multispeciality Hospital, Nalbari, IND

**Keywords:** intratracheal, intubation, trachea, complications, critical care, deglutition disorders

## Abstract

The ability to swallow and maintain the airway is a critical rehabilitation milestone for patients with swallowing disorders following prolonged tracheal intubation. Tracheostomy and dysphagia often coexist in critically ill patients and given the medical complexity analyzing the evidence to optimize swallowing assessment and management is challenging. It takes a holistic approach to dealing with issues in a critical care patient as we also need to deal with issues other than medical. We present a case of a 68-year-old gentleman who was admitted to the critical care unit following a double barrel ileostomy and had multiple complications and organ dysfunction requiring prolonged supportive management, tracheostomy, and mechanical ventilation. After recovering from primary illness and complications, he had a swallowing disorder (secondary dysphagia), which was managed successfully over the next month. The case highlights the need for screening, a multidisciplinary team, empathy, and effort as a part of a holistic management approach.

## Introduction

The incidence of secondary swallowing disorders in critically ill, post-tracheostomised patients is high (50-83%) [1]. Tube feeding, prolonged intubation, and prolonged mechanical ventilation are noted as risk factors for secondary intensive care unit (ICU) acquired dysphagia [2]. It adversely affects recovery, and patients experience adverse health outcomes like a delayed resumption of oral intake, aspiration and related pneumonia, and malnutrition. All these lead to increased length of stay, morbidity, and mortality; this culminates in a substantial economic burden [3,4]. Therefore, ICU-acquired dysphagia bears significant clinical importance. Initiation of oral feeding is a yet much-needed goal in managing such secondary dysphagia; the ability to swallow and maintain the airway is critical [5]. A tracheostomy tube (TT) makes it much more challenging to start oral feeding. Managing such dysphagia requires great effort and commitment from the healthcare staff with a multidisciplinary approach [6]. We present our experience, highlighting the need for screening, a multidisciplinary team, empathy, and effort as a part of a holistic management approach.

## Case presentation

A 68-year-old male presented with abdominal pain, recurrent vomiting, and not passing stool for three days following an alleged history of falls from the scooter. He has been enjoying good health despite a history of pulmonary tuberculosis 28 years prior. On arrival, he was conscious, afebrile, with a pulse of 116/min, blood pressure of 118/70 mmHg, and respiratory rate of 20/min. Ryle’s tube was inserted for gastric decompression. Emergency exploratory laparotomy, resection of gangrenous, and double barrel ileostomy were done.

The patient developed a fever and acute abdomen on the fifth postoperative day (POD). Despite antibiotics and nutritional management, bedside point-of-care ultrasound (POCUS) demonstrated bilateral pleural effusion. Hypoalbuminemia of 2.01 g/dL was noted at the time. On the sixth POD, POCUS also revealed significant abdominal collection; a re-exploratory laparotomy was done. Meanwhile, the patient developed septic shock and was on supportive therapy, including noradrenaline infusion (titrated to maintain the mean blood pressure of 60-65 mmHg) and mechanical ventilation. The patient was transferred to the critical care unit (CCU) postoperatively. He was weaned from noradrenaline by the sixth CCU day, and a percutaneous dilatational tracheostomy (7.5 mm internal diameter) was done on the 11th CCU day. He developed ventilator-associated pneumonia of *Pseudomonas aeruginosa* and *Proteus vulgaris* origin on the 18th CCU day. He was treated as per sensitivity and eventually weaned off the ventilator by the 23rd day. Daily mobilization and cuff pressure monitoring were continued as per standard CCU practice.

Enteral nutrition was provided through in-situ Ryle's tube from the third CCU day; oral feeding with semisolid food was tried on the 28th CCU day, but he could not swallow properly but coughed food material out through the TT. A differential of a swallowing disorder (dysphagia), vocal cord dysfunction, and tracheoesophageal fistula (TEF) were made and further evaluated [7,8]. A computed tomography scan ruled out TEF; vocal cord dysfunction was ruled out as he could produce an excellent cough. Further, his voice tone was fine when TT was occluded and cuff-deflated. We also fed colored yellow water to him, and immediately after swallowing, he coughed the colored water through the TT. However, when the cuff pressure was increased to >30 mm Hg before the feed started with oral water, he would inhale two times and rest three times so he could swallow it properly. We trained him to swallow using methods like an effortful swallow and made him perform jaw thrust, Masako maneuver, tongue strengthening exercises, and tasteful toffee (Kaccha aam - unripe mango), which was also provided [9]. The training continued for the next three weeks while feeds were given through Ryle’s tube.

**Figure 1:**
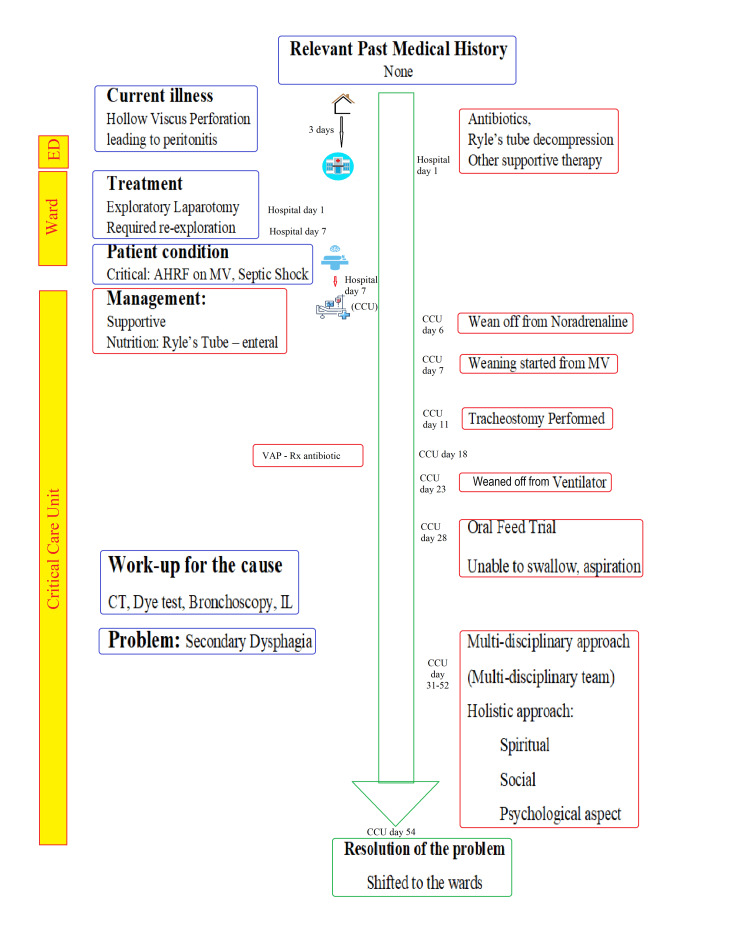
Showing the case timeline. AHRF - acute hypoxemic respiratory failure, CCU - critical care unit, CT - computed tomography, IL - indirect laryngoscopy, MV - mechanical ventilation, VAP - ventilator-associated pneumonia, ED - emergency department

Once we were sure that his swallowing had improved, as he did not cough vigorously following feeds, he had no episodes of desaturation, and his X-ray did not show any signs of aspiration and pneumonia; we decided to start him on semisolid oral feeds. We asked him to use a spoon for the food and take tiny portions of it, followed by multiple swallowing actions to ensure food transit into the esophagus and minimize or prevent inhalation of food. A professional nurse/critical care resident supervised all his oral feeds. We also sought the help of a speech therapist for further management. He wanted to read the Bhagwad Geeta, and that was arranged as well, which he read in his free time. Eventually, he was able to chew and swallow solids as well without aspiration by one month. He was then shifted to the ward, decannulated, and discharged later. The case timeline and events are summarized in Figure 1.

## Discussion

Secondary dysphagia following prolonged orotracheal intubation and tracheostomy is frequent. An understanding of the physiology of swallowing is critical for patient management. Swallowing is a complex neuromuscular process that enables the bolus' progression and transport, either liquid or solid, from the oral cavity toward the lower digestive tract [6].

Swallowing consists of phases: preparation and passage (push), oral, pharyngeal, and oesophageal phases. The pharyngeal phase starts with reflex activation of the pharynx. It activates the upper oesophageal sphincter, and the bolus enters inside, following which the esophagus contracts and the sphincter closes again. A proper bolus formation with a consistency comfortable enough to swallow is essential. Increased oral transit time also leads to loss of control of the bolus. Also, there was restricted upper excursion of the larynx during swallowing due to the TT in situ [6,10]. Further, the role of the tongue in deglutition is also crucial. The lingual complex has both local and systemic functions and can adapt negatively in the presence of abnormality and tend to lose proprioception and neuromotor coordination. The sense of taste also plays a fundamental role in the enteric system and the activation of the trigeminal lingual afferents. Chronic disease can cause motor dysfunction and alter the taste receptors’ activity [11]. All these lead to functional incoherence between the phases. Substances with enhanced taste, smell, texture, etc., can help increase the pre-swallow inputs to the cortex and brainstem, thereby decreasing the swallowing threshold [6]. In this case, we got him this tasteful toffee (Kaccha aam!!), and he could suck and swallow.

Lack of willpower, social support, and patient non-cooperations have been identified as critical barriers to rehabilitation [12]. The holistic management approach for the patient offered a comprehensive model for caring during the stay. Care is provided for the complete person concerning physical, social, psychological, and spiritual aspects [13,14]. A multidisciplinary team comprising the critical care physician (intensivist), otorhinolaryngologist, rehabilitation, critical care nurse, speech therapist, and dietician was involved in the comprehensive care. The critical team and the otorhinolaryngologist provided the TT care, and voice assessment, counseling, and management were done in coordination with the speech therapist. The critical care team and dietician took dietary management decisions, and psychological and social support was provided through regular counseling by nurses, critical care residents, and occasional counseling by the psychiatry team. We tried to get maximum patient cooperation with repeated counseling and boost his mental power by encouraging what he was habituated to before. Gita-Bhagawat (revered Hindu scripture) was arranged for him as he was a priest and read it regularly before he felt sick. It also took care of the spiritual aspect. Further, our team considered even the conventional practice to help to achieve swallowing reflex by providing non-medicated stuff like toffee (kaccha aam). Satisfying patients' desires for rewards such as kaccha aam and allowing them to read their favorite books are also compatible with sound medical practice.

## Conclusions

Diagnosing and managing secondary dysphagia following prolonged endotracheal and tracheostomy intubation requires a multidisciplinary medical approach. A holistic approach incorporating patients' participation, social interaction, and willpower improvement might aid in managing such cases.
